# Subjective Experiences of Mental Health Crisis Care in Emergency Departments: A Narrative Review of the Qualitative Literature

**DOI:** 10.3390/ijerph18189650

**Published:** 2021-09-13

**Authors:** Helena Roennfeldt, Marianne Wyder, Louise Byrne, Nicole Hill, Rory Randall, Bridget Hamilton

**Affiliations:** 1 Centre for Mental Health Nursing, University of Melbourne, Melbourne, VIC 3010, Australia; rory.randall@unimelb.edu.au (R.R.); bh@unimelb.edu.au (B.H.); 2 Research and Learning Network, Metro South Addiction and Mental Health Services, Brisbane, QLD 4122, Australia; marianne.wyder@health.qld.gov.au; 3 Menzies Health Institute Queensland, Griffith University, Logan Campus, Meadowbrook, QLD 4131, Australia; 4 School of Management, RMIT University, Melbourne, VIC 3000, Australia; louise.byrne3@rmit.edu.au; 5 Program for Recovery and Community Health, Department of Psychiatry, Yale School of Medicine, New Haven, CT 06513, USA; 6 Department of Social Work, University of Melbourne, Melbourne, VIC 3010, Australia; hilln@unimelb.edu.au

**Keywords:** mental health crisis, emergency department, subjective experiences, mental health emergency care

## Abstract

Mental health presentations to the emergency department (ED) have increased, and the emergency department has become the initial contact point for people in a mental health crisis. However, there is mounting evidence that the ED is not appropriate nor effective in responding to people in mental health crises. Insufficient attention has been paid to the subjective experience of people seeking support during a mental health crisis. This review aims to describe the qualitative literature involving the subjective experiences of people presenting to the ED during a mental health crisis. The method was guided by Arksey and O’Malley’s framework for scoping studies and included keyword searches of PsycINFO, CINAHL, Medline and Embase. A narrative analysis, drawing on the visual tool of journey mapping, was applied to summarise the findings. Twenty-three studies were included. The findings represent the experience of accessing EDs, through to the impact of treatment. The review found points of opportunity that improve people’s experiences and characteristics associated with negative experiences. The findings highlight the predominance and impact of negative experiences of the ED and the incongruence between the expectations of people presenting to the ED and the experience of treatment.

## 1. Introduction

The provision of mental health services, in Australia and internationally, has evolved from stand-alone psychiatric hospitals, to become mostly community-based [1]. From the 1990s, mental health services were “mainstreamed” whereby mental health and physical health were integrated, and psychiatric services became accessible via general health services [2]. This process of mainstreaming has fundamentally changed how people access emergency mental health services [3] and consequently, the emergency department has increasingly become the initial contact point for people in mental health crisis and the interface between community and mental health services [4].

Locally and internationally, mental health presentations to emergency departments have increased [5,6,7,8,9]. For example, the Australian Government reports that the number of mental health presentations rose from 136,026 in 2004/5 to 303,340 in 2018/9 and the proportion of mental health-related ED presentations increased from 2.9% to 3.6% of all presentations, from the years 2004/5 to 2018/19 [10]. These statistics, even so, are likely to under-report mental health-related presentations to the ED and only report on ED presentations with a principal diagnosis of mental illness [10]. Comparable rates of mental health presentations were found in the UK and Canada [11], with a higher rate of 10% in the US [12]. Alongside these increases are reports of inaccessibility to support [13]. This is significant given that EDs have become a focal point for suicide treatment interventions in the US, UK and Australia [14].

Mental health presentations create tensions for EDs by disrupting the treatment norms and flow of the ED, evident in the rising rates of “psychiatric boarding”—referring to people waiting in the hallways and ED rooms for a mental health inpatient admission [15,16,17]. Individuals presenting to the ED due to their mental health often experience particularly long wait times compared to physical health presentations [18]. Moreover, the often over-crowded, over-stimulating and time pressured environment, and limited qualified mental health staff increasingly suggest the ED is not an effective place for individuals in a mental health crisis to receive support [13,19].

The experience for people presenting to the ED with a mental health presentation is further complicated by higher rates of restrictive practices compared to people who present due to their physical health [20]. Restrictive interventions are administered through coercive means that limit autonomy and include involuntary hospitalisation, physical and mechanical restraint, and forced medication [21]. Consequently, the risk of physical and psychological trauma is significant. The Australian College of Emergency Medicine reports that people experiencing a mental health crisis are up to 16 times more likely to arrive at the ED by police than people with medical conditions and nearly twice as likely to arrive at EDs via ambulance [18]. The increasing use of ambulance and police has been found to escalate the presenting situation, intensify distress, and the involvement of police has been linked to increasing public stigma and criminalisation of mental illness [22].

Recovery-oriented service delivery has been widely adopted within mental health policy and has been core to mental health reform and better outcomes for people accessing services [23,24]. Recovery-oriented service delivery is a person-centred approach that is responsive to individual needs and empowers people to participate in decision making [25]. Criticisms have been raised regarding the extent that recovery-oriented practice is evident and implemented within mental health practice broadly [26,27]. Overwhelmingly, the biomedical model still dominates responses to mental health presentations within the ED. The biomedical approach, which emphasises pharmacological approaches to treating symptoms, has faced critique for being paternalistic and not adequately taking account of psychological and social contributors to mental illness [28,29]. This is significant given the evidence that the way people are supported in a mental health crisis can critically impact their recovery [30].

There has been limited research exploring individuals’ experiences of emergency departments. In 2017, Carstensen and colleagues sought to synthesise qualitative evidence of subjective experiences of the ED using a CERQual review [31]. Findings from this review identified the effects of staff relationships, wait times and the physical environment on individual’s experiences of stress and discomfort. The review, however, was limited to nine studies and blended mental health and physical health presentations to the ED. The search was also limited to certain diagnosis and did not cover the breadth of mental health presentations to the ED. Therefore, an updated review of qualitative studies focused specifically on mental health presentations and inclusive of all mental health presentations is warranted, to include diverse presentations to the ED. Since this review by Carstensen and colleagues, several significant studies have also been published that are important to include. The findings in the current systematic review further expand upon previous work by building a journey map of individual experiences of the ED from the initial point of entry to the ED through to exploring the impact of their ED experience. The review examines the literature to answer the following question: what have been the subjective experiences of mental health crisis responses in EDs and what factors influence the outcomes of support received during a mental health crisis?

The review was informed by the following definitions and understanding of mental health crisis and emergency mental health care:

Mental Health Crisis: a state where an individual becomes overwhelmed, and their usual coping mechanisms are not adequate, leaving them with disorganised and intolerable thoughts and life processes [32]. This state can include suicidal ideation, extreme panic, feeling overwhelmed with life situations and/or symptoms of illness, as well as injury and illness that arise from a mental health diagnosis [33].

Mental Health Emergency Care: an immediate response by one or more individuals to the acute distress experienced by another individual, which is designed to ensure safety and recovery and lasts not longer than 1 month [34], within the context of the ED or as part of access to the ED.

## 2. Materials and Methods

The review was guided by Arksey and O’Malley’s methodological framework for scoping studies [35] and follows the Preferred Reporting Items for Systematic reviews and Meta-analysis extension for scoping reviews to ensure rigour (PRISMA-ScR) [36,37]. A narrative synthesis methodology was applied to summarise, explain and interpret findings [38]. Recognising the time sequence of ED experience, findings have been presented based on journey mapping to depict the series of events that shaped the subjective experience of the ED [39].

### 2.1. Search Strategy

Potentially relevant publications published between 1 January 2000 through to 1 April 2020 were identified by searching the following electronic databases: PsycINFO, CINAHL, Medline and Embase. The search strategy consisted of keywords and related terms for subjective experiences, mental health care and emergency services. These terms were informed by the research question and developed in consultation with an academic librarian. Table 1 outlines the search terms or subject headings (and related concepts, depending on the database) that were used:

### 2.2. Inclusion and Exclusion Criteria

The inclusion criteria were formulated using the PICOS (Participant, Intervention, Comparison, Outcome and Study design) [40]. Studies were included that were available in English if they met the following criteria.

#### 2.2.1. Study Design

All qualitative primary research was considered for inclusion, regardless of study design. Studies were excluded if they were quantitative, whether epidemiological/descriptive or reporting interventions. Non-primary research, such as commentaries and protocols, was excluded. Reviews of the literature were also excluded but first screened for pertinent primary studies that they have included.

#### 2.2.2. Participants

The studies’ populations included people attending an ED with a mental health crisis (including presentations involving references to suicide, substance abuse, psychosis, and personality disorders). Studies of participants under 18 years of age were excluded. For studies with a mixed population of participants, including people attending the ED, their families and ED staff, the study was included if the outcomes pertaining to experiences for people attending the ED could be extracted. Studies were excluded if they focussed on general medical presentations by mental health consumers. The review only included studies set in an ED or studies that involve emergency services where the individual is taken to the ED. Studies reporting on mobile crisis or alternative crisis responses or any emergency services that did not result in a presentation to the ED were excluded.

#### 2.2.3. Intervention

Mental health emergency care in this review refers to service or treatment delivered to people experiencing a mental health crisis in the ED. The intervention was provided by ED staff or security and emergency services in any ED setting, in any geographical region.

#### 2.2.4. Comparison

Qualitative studies with or without a comparator group were included.

#### 2.2.5. Outcome

Studies reporting on qualitative data regarding people’s experiences of EDs were included. Studies reporting solely on satisfaction surveys using quantitative measures were excluded if they did not include qualitative data. Standardised satisfaction questionnaires and surveys have been criticised for their inherent bias in embedding positive satisfaction in the design of the evaluations and consequent lack of accuracy in reflecting subjective experiences [41,42]. Studies were excluded if they described staff perspectives or both consumer and clinicians’ perspectives, and consumer perspectives were not distinguishable.

### 2.3. Study Selection and Data Extraction

A multi-staged screening process was used to determine the eligibility of articles. Three members of the research team screened articles for inclusion (H.R., R.R., M.W.). This involves a systematic process to filter for duplicates automatically (in Endnote) and manually, followed by screening titles and abstracts of potentially relevant studies. Relevant articles identified in the title and abstract scans underwent a full-text review to confirm the articles’ eligibility for the study. Any disagreements were discussed and resolved by the researchers. The reference lists of included full-text sources were also screened to identify any potentially relevant publications for inclusion.

The documents for final review were imported into the QSR International qualitative software package NVIVO (2018). A general inductive approach was adopted to analyse the findings of the included studies, identifying themes emerging across the data [43]. Extracted data were examined and re-examined, guided by initially identifying lower- and then higher-order theming [44]. The research team discussed emerging themes until all data were accounted for and the most stable themes were able to be identified [45,46].

The experiences and themes were then mapped along a journey map. These maps are commonly used in healthcare, to depict the service experience of people accessing services and incorporate physical events, the experience of receiving treatment as well as the subjective experience [39]. Journey maps allow the identification of mitigating factors (or points of opportunity) that were found to improve people’s experience. They also allow pain points (or aspects that were associated with negative experiences) to be exposed for people accessing the ED [47].

## 3. Results

**Figure 1:**
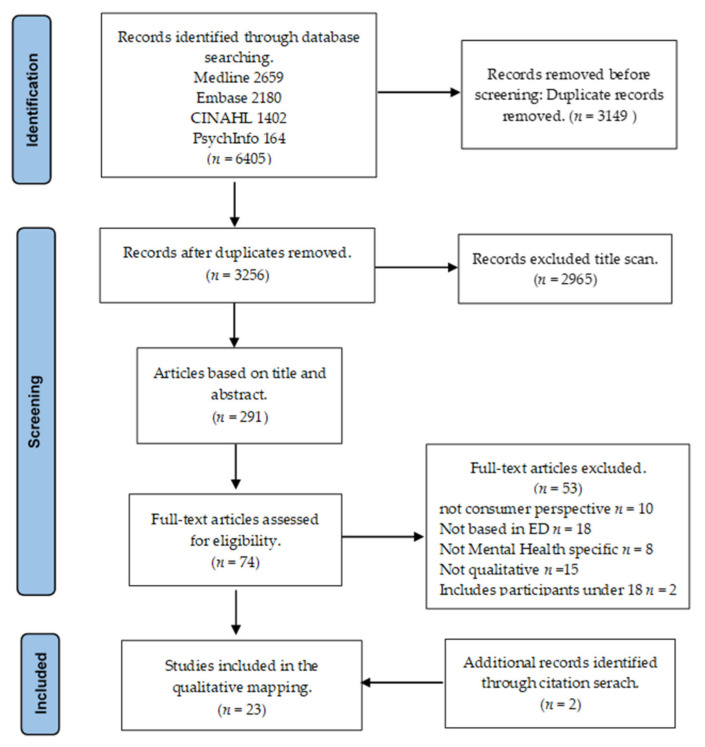
PRISMA flow diagram.

The search across the four databases identified 6405 titles, with 3256 remaining after 3149 duplicates were removed. All titles of retrieved articles were scanned, and 2965 articles were removed. The abstracts of the remaining 291 articles were reviewed and a further 217 were removed. The full texts of these 74 articles were then read, and a further 53 were excluded. An additional two articles were identified through other sources, leaving a total of 23 articles included in the final review; the PRISMA diagram is presented below [48] in Figure 1.

### 3.1. Characteristics of Studies

Of the 23 studies, 7 were conducted in the US, 6 in Australia, 5 in Canada, 4 in the UK and 1 in Belgium. All involved qualitative data collection with methods, such as case study; focus groups; interviews; and/or surveys. Eleven studies used a mixed-method approach of both surveys and interviews, surveys and focus groups, or surveys involving both quantitative and qualitative data. Four studies applied secondary analysis to existing data. One study involved an in-depth case study of the experience of the author. Five of the studies included perspectives of both consumers and professionals or consumer and carers; however, for this review, only the consumer perspectives reported are included in the analysis. Three articles were from the same authors and represented the same study [49,50,51].

Five of the studies investigated specific mental health populations. One study included individuals who attended the ED following a suicide attempt [52]; two studies focused on “frequent presenters” to the ED [53,54]; one study focused exclusively on individuals diagnosed with borderline personality disorder [55]; three studies specified experiences of coercive and restrictive approaches [56,57,58]; two studies focused on experiences with mental health staff in ED [59,60]; and the two remaining studies compared experiences of consumers who had used both ED and a community crisis service [61,62]. Studies identified the reasons for presentations most often as suicidal or self-harming behaviour [50,55,57,61,63,64,65], mental health problems or symptoms [50,57,58,64,66,67], substance use and co-occurring conditions [55,57,61,64,68], seeking connection or admission [53,55,64], negative or violent behaviour [55,58], or social determinants [58]. Three studies identified the inclusion of participants who reported it was their first mental health presentation to the ED [56,59,63,69]. Further details of included studies are available in the data extraction table in Appendix A.

### 3.2. Narrative Analysis of the Subjective Experiences of People: The Journey Map of Experience in ED

The experiences of the ED were grouped in the following broad themes: access to ED; interaction with staff; treatment experience in ED and outcome of ED presentation. Each major theme contains multiple subthemes. Table 2 provides the occurrence of each of the major and subthemes within studies.

**Figure 2:**
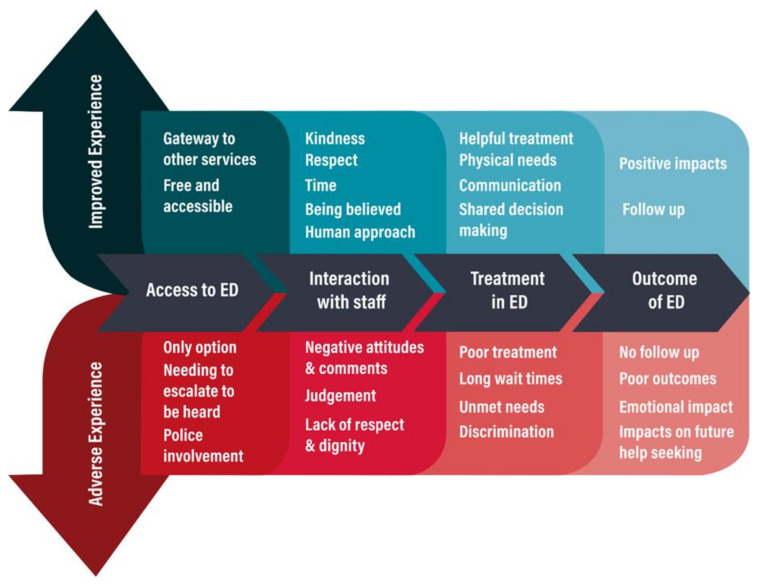
Journey of experiences in ED.

A detailed description of each of the major and subthemes is provided as part of the journey from entry to the ED through to the outcome and impact of the ED. Using the language of journey mapping, mitigating factors or points of opportunity for participants are first provided, followed by “pain points” or factors that led to more adverse experiences. Figure 2 is a representation of these themes on a journey map.

#### 3.2.1. Access to ED

Studies described ED as free and accessible, but also as the only option and last resort. Access to the ED was further shrouded in conflicting perceptions of the appropriateness of presentations at the ED.

##### ED Accessible and Appropriate

Studies reported that the ED was perceived by participants as accessible and an appropriate place to seek support for their mental health crisis [50,53,54,55,61,63,65,67,68]. Community and primary health services were also seen to endorse ED as an appropriate place of support by advising people to call emergency services and encouraging access to the ED [54,55,67]. Many studies also described participants with complex and persistent needs that could not easily be resolved by community services [50,53,54,55,65,68]. ED also served as a gateway to other services and provided participants with faster access to services, particularly drug and alcohol services [54,55]. The severity of suicide attempts and self-injury further reinforced that the ED was a necessary and appropriate option [54]. However, it was evident from the studies that there was a clash of viewpoints regarding appropriateness for participants and the ED staff [53,54]. Similarly, participants described the need to escalate in frank expressions of distress in order to be heard or wait until the crisis was severe enough to be noticed [65,68,70], even though the majority of participants arriving at the ED with a mental health presentation were assessed as urgent [53,54,64,68].

##### Only Option

Studies identified the ED as unavoidable and the only available option [53,54,55,58,61,63,65,66,67,68,70,71], or a last resort when other options had been exhausted [54,55,58,70]. The majority of participants in studies were taken to the ED by family, ambulance or police and did not access the ED voluntarily [52,53,54,55,57,66]. Involuntary presentations to the ED were also linked to the experiences of force used by police, increasing fear and humiliation [52,54,61,62,65,68].

#### 3.2.2. Interactions with Staff

Many studies reported variable (very good to very poor) interactions with ED and emergency staff, even in the same ED.

##### Positive Interactions with Staff

Positive experiences with staff included descriptions by participants of being listened to, given time, taken seriously and shown compassion [50,51,52,59,62,63,67,69,70]. Favourable comments were provided by participants about staff advocating on their behalf [68], providing hope [67,71] and personally offering to provide follow up [63]. Participants also appreciated staff who knew them and their story from other occasions [53]. Studies described human aspects of care that could, in part, override the negative effect of the physical environment and negative impacts of treatment received in ED [49,50,57,59,62,63,67,68,70]. Human qualities, such as kindness and someone to listen, were also seen as reducing the need for restraint [62].

##### Knowledge and Expertise of Staff

Participants valued staff who had mental health knowledge and training [50,51,52,59,60,66,69,71], such as mental health liaison nurses, who were seen to recognise participants’ needs and respond more effectively [50,60,69]. However, in many studies, participants felt that the staff lacked expertise and training in mental health and substance use issues [57,59,63,68].

##### Judgemental Attitudes

In most studies, participants also reported experiencing judgement from staff in the ED. These ranged from laughing to inappropriate comments [50,51,52,53,54,55,61,63,65,67,68,70,71]. Studies of participants who presented frequently to the ED described predominantly negative interactions and impatience from staff [53,54]. In a similar manner, studies also reported participants feeling judged by ambulance and police who had accompanied them to the ED or who were present at the ED [53,61,65,68].

#### 3.2.3. Experiences of Treatment

Although there were some accounts of positive experiences of treatment, most participants described unsatisfactory treatment and unmet needs in the ED.

##### Positive Experiences of Treatment

There were some studies where participants identified favourable experiences and satisfaction following their presentation to the ED, including adequate attention to physical injuries and helpful mental health care [50,51,57,63,64,66]. Positive outcomes also related to the role of mental health staff in providing specialised treatment [59,60,69].

##### Long Wait Times

A pervasive finding across the studies was that participants reported experiencing long waiting times in ED, exacerbating participants’ distress [50,51,52,54,55,56,57,58,59,61,63,65,66,67,68,69,70,71]. Conversely, timely support and providing information regarding wait times improved the perception and satisfaction with the ED service [67,69].

##### Physical Space and Lack of Privacy

The ED was described as overstimulating [50,59,61,65,68] and unwelcoming [54,61,67,70]. Instead, a calm, soothing environment [67] and comfort were essential attributes relating to physical space that were sought after [62,67,68]. A lack of privacy was also noted in many studies [49,50,51,53,59,61,62,63,66,67,68,69,70,71], resulting in many participants describing feeling vulnerable [59,63,70].

##### Unmet Needs

Studies described the negative impact of not having basic needs met, such as food, drink and bathroom facilities [55,62,65,66,67,68,70,71], and the distinct contrast and positive impact when being provided food, a blanket, clothes [62,67] and access to the bathroom, notably for participants while they were in restraints [67]. One study reported that some participants preferred to see a female staff member, but this was not always possible [66]. Cultural needs were also identified by some studies as unmet within the ED [52,63].

##### Poor Treatment

The majority of studies reported very poor satisfaction and negative experiences of participants regarding their treatment in the ED [52,53,54,55,56,58,59,61,63,64,65,67,68,70,71]. Studies reported medical needs not being addressed or cursory or unsatisfactory checking of physical symptoms [62,68]. Other studies describe invasive and irrelevant medical tests [51] and intrusive questioning [59,70]. However, some studies show participants did report satisfaction with attendance by staff to physical injuries [49,61,66].

Studies frequently reported a lack of response to suicide attempts and self-injury [52,55,56,58,62,65,67,68,71]. Studies similarly reported a lack of mental health and emotional support [53,59], and a perception that help was not available when needed [53,59]. Minimal time spent with participants led them to wonder whether the ED staff understood their needs or simply based their treatment on pre-existing ideas of the care required [49]. Unsatisfactory treatment was also related to lack of information and involvement in decision making [49,52,56,63,64,67,68]. In comparison, the perception of choice and shared decision making was linked to more positive experiences of EDs [52,56,62,67].

##### Discriminatory Treatment

Studies revealed that participants did not consider their mental health needs to be treated the same as physical health presentations at the ED [49,58,61,65,67]. Having a physical complaint was seen to legitimise the presentation to the ED and participants felt that ED staff would be more likely to accept their need for emergency care [53,55,61,65,70]. In particular, studies with participants who presented frequently [61,62,65] and those diagnosed with Borderline Personality Disorder described feeling discriminated against by staff and considered themselves to receive worse treatment than other mental health presentations [52,55,59,60,70]. Similarly, studies involving participants who were homeless [70], presentations involving self-harm [55] or those with alcohol and substance use reported feeling judged and unwelcome at the ED [53].

##### Coercive and Restrictive Practices

Restrictive practices were widely experienced as harmful [52,53,56,58,61,62,65,67], with studies describing the experience as terrifying, dehumanising and isolating and participants detailing experiences of physical, verbal and sexual abuse [58,67]. Studies describe seclusion and restraint as overused [67] and participants reported adverse and sometimes severe side effects from forced medication [52,67]. Experiences of restraint were compared to being in prison [61] and studies reported participants being unaware of their rights [58,65].

Factors that made the experience less traumatic included participants having a support person with them who could explain what was happening and advocate on their behalf [67]. In a small number of studies, participants reported that there were times restraint was appropriate and that being involuntary allowed them faster treatment [62].

#### 3.2.4. Impact of ED

Positive outcomes of the ED included staff providing meaningful follow-up and appropriate treatment. However, most studies described the negative impacts of the ED.

##### Follow up

Follow up was valued by participants and included information, referrals and being told they could return if they needed to [50,51,58,63,65,66,69]. Being handed leaflets was considered the least useful by participants [63,65]. However, it was common for studies to describe participants being discharged without follow up [49,50,53,55,59,61,68], or discharged without mental health treatment [52,54,70].

##### Poor Outcomes

Poor outcomes were evident in studies reporting cases of participants leaving the hospital without being seen [59,65,68,70,71] and re-presentations to the ED following the continuation or exacerbation of distress [53]. Studies also described physical injuries resulting from restraint procedures [58].

##### Negative Emotional Impact

Studies describe participants’ experiences of shame and guilt as a result of their presentation to the ED [50,52,54,55,56,68]. Experiences of being perceived as misusing the ED left some participants feeling dismissed and humiliated [54,55,61]. In other studies, the emotional impact included fear and feeling punished [52,58,65,68]. Experiences of restraint exacerbated existing mental health conditions and had lasting psychological consequences [52,57,58].

##### Re-Traumatisation

Studies described participants experiences of previous trauma and how this shaped their experience of treatment in the ED [52,53,56,61,68]. Restrictive practices in the ED re-traumatised participants and caused them to relive earlier experiences of being abused [58]. Studies also revealed how seemingly routine care-related requests, such as being asked to wear a hospital gown, could be perceived differently by individuals with a history of trauma and viewed as a lack of understanding of emotional vulnerability [61].

##### Future Help-Seeking

Negative experiences of coercive practices within the ED impacted future help-seeking [57,62,67], with one study revealing that over half of the participants who experienced restraint and seclusion stating that they would be unwilling to seek help in the future [67].

## 4. Discussion

This review provides an overview of the qualitative literature on mental health presentations to emergency departments. The review identified 23 studies. Most studies used small sample sizes and reported on subjective experiences as a subset of data collection. The findings have been depicted as experiences along a journey from accessing care in the ED through to the impact of the experience of the ED. While the ED was often considered the only option available for people, it was clear that the ED was generally not appropriate based on the experiences of the treatment available for the mental health needs. Consequently, many people experienced negative impacts, including unmet physical and psychological needs.

The findings regarding access to the ED reflect gaps in community mental health service provision internationally, with existing services unable to meet the complex needs of mental health consumers who are presenting to the ED [72]. This reinforces the reality that accessing EDs for treatment is frequently unavoidable, given the current service context and lack of acceptable alternatives [19]. Yet, the findings also identify a clash in the perception of appropriateness of presentations held by participants and ED staff. Increased demands on EDs and concerns regarding public health system sustainability have increased questions of the appropriateness of presentations, particularly concerning frequent presenters, and these concerns may be reflected in staff views [73].

Similarly, there is a mismatch of expectations between what constitutes appropriate treatment responses, with participants often seeking hospital admission, and ED clinicians seeing diversion to community services as the more successful outcome [11,68]. The lack of clarity about what is needed, and effective treatment requires further investigation, to better understand the needs of people seeking support and to design effective alternative services. These findings further reflect systemic issues in mental health services, with responses to crisis as only a default arrangement in the current funding climate, not an active and planned service model or response [19,63].

The questions of appropriateness of presentations and the perceived lack of legitimacy of mental health presentations apparent in the findings may have particular significance for the continuing need to address stigma [74]. It also points to societal perceptions of those deserving of care, as opposed to those who are not [75]. Depictions of those worthy of care may reflect a broader systemic response that is understood by discrepancies in wait times and staff attitudes towards mental health presentations. Similar to mental health presentations, satisfaction with treatment for physical health presentations within the ED is linked to perceptions of the quality of care received [76]. Despite similarities, mental health consumers’ experiences of emergency departments are likely more impactful, as indicated by findings of shame, humiliation and feeling punished. Future research and comparisons between experiences of individuals who present with a mental health crisis and those who present with a physical health crisis could reveal essential similarities and distinctions.

The physical environment of the ED was also found to be a limiting factor for individuals in receiving support during a mental health crisis. EDs were considered overly stimulating environments and lacking in privacy [77,78,79]. The emergency department’s current built environments contrast with recommendations that the ED provides a quiet and non-stimulating environment (Mental Health & Drug Alcohol Office, 2009). Curiously, mainstreaming’s goal was to reduce stigma by integrating mental health within general medical services, yet in some studies, participants recommended a separate access area from the main ED to avoid stigma [49]. This mirrors recent ED reforms that have focused on providing specialised care in mental health emergency departments or separate waiting areas [80].

Although negative experiences predominated, some positive experiences of treatment and interactions are noted, including common experiences of EDs being accessible and of people experiencing some positive interactions with staff. Findings highlight the importance of the quality of the helping relationship, with positive interactions protective against the negative experiences of care in the ED. Mainly because of the stigma still associated with mental health concerns, experiences of kindness and compassion are likely to be even more highly valued by consumers seeking support or being subjected to involuntary treatment. In contrast, negative experiences exacerbated the negative impact of these experiences, and serve as a disincentive to further accessing support [74]. It is striking that staff responses in the ED are often incongruent with contemporary recovery-oriented mental health care that recognises the expertise and autonomy of consumers [4]. Negative attitudes held by some staff may be related to staff lacking opportunities for training and consequently, having limited skills to support the needs of mental health consumers [66,68,81]. These findings bolster the importance of mental health training for ED staff, including training in recovery-oriented practices, and emphasise the fundamental role of interpersonal skills including communication and empathy alongside technical or clinical skills [82].

Critically, findings regarding the use of seclusion and restraint detect potential breaches to the human rights of participants [83,84]. The use of seclusion and restraint as seemingly acceptable management tools for people with a mental health diagnosis underlines the difference between physical health presentations [85]. Findings from this review parallel the existing literature, revealing the potential for traumatisation, re-traumatisation and experience of shame, compounding fears that seeking treatment will result in being held involuntarily [86].

The narrative analysis highlighted important considerations for the impact of experiences of seeking care in the ED. Overall, there was a predominance of distress and discomfort experienced by participants as a feature of the care received, in addition to the distress that brought them to the ED. Participants often reported judgement, disrespect and disregard when interacting with ED staff, which negatively impacted their perception of the care they received, deterring future help-seeking. These factors are significant given the increasing rates of mental health presentations to the ED, lack of alternatives and increasing point of interaction for the treatment of people in suicidal crisis [14]. Subjective experiences and preferences for mental health emergency care are important, given consumer experiences could play a deciding role in determining the best approach to support individuals in a mental health crisis. There have been calls for greater inclusion of consumer voices in health services planning and some recognition in policy [87,88,89]. The inattention to subjective experiences and preferences within the research also raises a philosophical question as to why the perspectives of people who access EDs are being neglected. Individuals’ experiences and expectations in accessing emergency care for mental health require further investigation to inform service reform and decision making. These findings foreground the importance of human connection and autonomy for people in mental health crises and highlight the need to develop alternatives to reduce negative impacts and increase the potential for crisis intervention to aid recovery. Current treatment available in the ED does not adequately address the complex challenges associated with mental health presentations. Together, the findings from this study call for radical change in the practices of emergency mental health care and re-envisioning current models of mental health emergency care delivery. Because of its dominant position in current mental health service delivery, the ED is strategically positioned to make important contributions in shaping the future of mental health care.

### 4.1. Strengths and Limitations of This Study

This review establishes a baseline understanding of the experiences of mental health presentations in the ED. This is a timely and vital topic required to support ED reforms and inform alternative models of mental health crisis care. The review used a rigorous study design; however, a limitation is the potential to miss relevant articles given that subjective experiences are not always separated from other outcomes.

The studies included are limited to the UK, Europe, Canada, and Australia and given differences between health systems, the results may not be generalisable to other countries. These countries have previously been found to have comparable health systems, which strengthens the collective results from this review [11]. However, the lack of non-Western countries is a limitation of this study and future research should focus on broadening the search and including more diverse databases such as “Lilacs” to increase the potential inclusion of the perspectives of people’s experiences of mental health emergency care in these countries.

Furthermore, the studies were also limited in mostly describing the experiences of participants who had multiple presentations to the ED, and this may not reflect participants with infrequent presentations or presenting for the first time. An epidemiological study by Barrett and colleagues found that 40% of the mental health presentations to emergency departments are first time presentations [11]. Given the complexity and the heterogeneous needs of people presenting to the ED with a mental health crisis, implementing interventions and alternatives to emergency departments without understanding the subjective experiences and preferences of people in a mental health crisis, including participants who presented for the first time or infrequently, could jeopardise the success of articulated international and national reform directions and priorities [72,90,91].

### 4.2. Implications and Future Directions

Presently, mental health emergency care is at a critical point with rising investment into ED reforms and emerging alternatives to the ED within community services. It is essential at this stage of system reform that the unique insights provided by first-hand experiences of treatment are understood and utilised to enhance existing clinical practice and inform reforms. Pervasive adverse experiences of people accessing EDs for mental health presentations and a lack of parity between physical health and mental health presentation support the need for investment into ED alternatives and underline the need for increased understanding of the impacts of the ED for mental health consumers. Significantly, negative experiences of treatment led to experiences of shame and fear and impacted future help seeking.

Although a relatively small number of positive experiences of treatment and interactions with staff were reported, there is potential to use this study to recognise the positive impact of individual acts of compassion and to reposition the importance of relational aspects of mental health crisis care in the ED, and the enduring need for crisis services to be as readily accessible as ED. However, the push for rapid crisis responses may be impacted by wider demand for emergency health services and economic rationalisation, which highlight the need to promote the importance of subjective impacts beyond clinical outcomes and financial benefit. Service gaps highlighted by consumers in this study mirror policy objectives of accessibility, timely support and equity of care and reinforce the need for improved mental health crisis care to achieve better outcomes for people accessing crisis services.

Overall, our systematic review raises the question of the sustainability and long-term reforms needed to develop effective responses for people experiencing a mental health crisis. Future research should also evaluate the impact of receiving mental health crisis care, to ensure that the adverse experiences identified in this review are minimised and addressed in current and future mental health emergency care. This review was conducted as the basis for a larger empirical study of subjective experiences of mental health crisis care.

## 5. Conclusions

This review of people’s experiences of MH care in EDs underscores the dire impact on people in MH crisis because of the shortfall in expertise and resources, both in the community and in EDs. Future studies should examine which components of mental health emergency care make the greatest contribution towards improving outcomes for people in mental health crises. This systematic review highlights significant gaps in the current literature regarding understanding people’s experiences of mental health emergency care. The lack of well-designed and lived-experience-informed research on people’s experiences of mental health crisis and effective assistance is troubling. Understanding the changes needed for ED models and staff to support people in a mental health crisis is an essential next step in the research.

## Figures and Tables

**Table 1:** Search terms used in the search strategy.

Person/Population AND	Subjective Experience AND	Characteristic AND	Environment
Consumer * or “service user *” or patient * or client *	experience * or subjective or perception * or perspective * or qualitative or preference * or satisfaction	psychiatric * or “mental health” or “mental Illness” or “mental health crisis” or psychological* or “mental disorder” or “emotional trauma” or “psychological trauma” or “emotional distress” or “psychological distress” or “mental distress” or suicidal or “suicide attempt” or “self-harm” or “substance use” or “substance abuse	“emergency department *” or “emergency service *” or “accident and emergency” or ambulance or police

Note. Use of wildcards, such as asterisks (*) and quotation marks (‘’) were tailored to databases. In the table quotation marks are used to search for the exact phrase and * is used as the truncated symbol to search for plural use of the term and variant spelling.

**Table 2:** Major and subthemes, and the studies in which they occur.

Theme	N Studies	Studies
Access to ED		
Accessible and appropriate	10	[50,53,54,55,61,63,65,66,67,68]
Only option	13	[53,54,55,57,58,61,63,65,66,67,68,70,71]
Interactions with staff		
Positive interactions with staff	14	[49,50,51,53,59,60,62,63,66,67,68,69,70,71]
Knowledge and expertise	9	[50,51,59,60,63,66,68,69,71]
Judgemental attitudes	14	[50,52,53,54,55,60,61,63,64,65,67,68,70,71]
The mitigating effect of staff	9	[49,50,52,56,57,59,62,63,67,70]
Experience of treatment		
Wait times	18	[50,51,52,54,55,56,57,58,59,61,63,65,66,67,68,69,70,71]
Poor and inadequate treatment	16	[51,52,53,54,55,56,58,59,61,62,63,64,65,67,68,70]
Positive experiences of treatment	6	[50,51,57,63,64,66]
Discriminatory treatment	15	[50,52,53,55,56,58,59,60,61,62,65,67,68,70,71]
Unmet needs	11	[50,51,55,59,63,65,66,67,68,70,71]
Restrictive practices	8	[52,53,56,58,61,62,65,67]
Privacy	14	[49,50,51,53,59,61,62,63,66,67,68,69,70,71]
Physical environment	10	[50,57,59,61,62,65,67,68,70,71]
Outcome of ED		
Poor outcomes	7	[53,58,59,65,68,70,71]
Negative emotional impact	8	[50,52,54,56,57,59,66,68]
Impact on future help-seeking	3	[61,62,67]
Positive outcomes	3	[50,51,57]
Follow Up	15	[50,51,52,55,58,59,61,62,63,65,66,68,69,70,71]

## Data Availability

No new data were created or analysed in this study. Data sharing is not applicable to this article.

## Appendix A

**Table A1:** The data extraction table of included studies.

Author	Year	Title	Journal	Study Design	Country	Participants	Data Collection	Data Analysis	Main Findings	Aim	Limitations/Strengths
[67]	2003	What do consumers say they want and need during a psychiatric emergency?	Journal of Psychiatric Practice	Mixed methods	USA	*n* = 59 Participants with a diagnosis of mental illness and at least one emergency service experience involving medication, seclusion, or restraint	Survey and focus group Surveys were also conducted with mental health professionals	Thematic analysis	Mostly poor experiences with staff Variable experiences with treatment and addressing needs Adverse experiences with medication, seclusion and restraint and impact on future help-seeking	To better understand consumer experiences and preferences	More women than men Mostly Cauca-sian Length of time elapsed since ED on average eight years
[69]	2002	Patient feedback on liaison mental health care in A&E	Nursing Times	Mixed methods	UK	*n* = 17 Participants with experience of ED and hospital	Semi-structured interviewsUser satisfaction survey—including open-ended questions	Thematic analysis	Valued having staff with MH knowledge Variable experiences of treatment Long wait times	Explore experiences of consumers with MH liaison staff	Use of satisfac-tion survey with qualita-tive responses difficult to de-termine
[64]	2005	Service expectations and clinical characteristics of patients receiving psychiatric emergency services	Psychiatric Services	Mixed methods	USA	*n* = 82 Participants with previous admissions	Self-report survey	Content analysis	A mismatch between consumers expecta-tions and services available in ED	Explore the use of the psychiatric emergency department and expectations of services availa-ble in the emer-gency department	Unique survey form and grouping of content for analysis of open-ended questions
[52]	2006	Consumer and family experiences in the emergency department following a suicide attempt	Journal of psychiatric practice	Mixed methods	USA	*n* = 465 Participants who had visited the ED following a suicide attempt. Family members (*n* = 254) members	Surveys Separate anonymous surveys were created for two groups. Survey had mostly yes/no responses with one open-ended question	Thematic analysis of the open-ended question	Long wait times Mixed results re-garding interactions with staff Suicide attempts were not taken seriously Cultural needs not always addressed Did not feel listened to or provided with information	To understand the separate experiences of consumers and family members in the Emergency Department following a suicide attempt	Focus on sui-cide attempts Unable to compare sur-vey results between con-sumers and carers Majority female Majority Cau-casian
[68]	2007	Emergency department from the mental health client’s perspective	International Journal of Mental Health Nursing	Qualitative	Canada	*n* = 27 Participants (consumers) Family members (*n* = 7) and stakeholders (*n* = 5)	Focus groups (separate focus groups for consumers)	Thematic analysis	ED was the only option Long wait times Discriminatory treatment Lack of privacy Physical and basic needs not met Importance of the relationship with staff The criminalisation of mental health	To determine consumer and their family satisfaction with care received in ED	Participants self-selected Most had numerous experiences of ED Lack of diversity of culture and from regional areas Emphasis on the role of the PEN
[53]	2018	Exploring the experiences of persons who frequently visit the emergency department for mental health-related reasons	Qualitative Health Research	Qualitative	Canada	*n* = 10 Participants who had 12+ ED visits within a 1-year time frame	Interviews	Thematic analysis using	ED was appropriate and unavoidable Participants felt dis-missed Prejudicial treatment Physical complaints legitimised the ED visit Lack of follow up Being known was both positive and negative Variable experience with staff Seeking connection	Explore the experiences of persons who frequently visit the emergency department (ED) for mental health-related reasons	Focus on fre-quent presenters Small sample size. Varying times since accessing ED Majority diagnosed with BPD; also co-occurring substance use disorder
[59]	2006	Service users and other stakeholders’ evaluation of a liaison mental health service in an accident and emergency department and a general hospital setting	Journal of Psychiatric and Mental Health Nursing	Qualitative	UK	*n* = 17 Participants (consumers) Professionals (*n* = 30). Professional stakeholders include A&E nurses, community M.H. professional and police	Interviews	Thematic content analysis	MH nurse improved ED experience Discomfort with phys-ical environment of ED	Explore what is important to service users and professional stakeholders in the provision of ED service	Random sam-pling, but small sample size Emphasis on the role of the MH liaison nurse Included first- time presenta-tions
[63]	2019	Satisfaction with emergency departments and other mental health services among patients with mental disorders	Healthcare Policy	Mixed Methods	Canada	*n* = 328 Participants who had presented to ED.	Surveys (standardised and qualitative items) and Interviews	Convergent mixed methods design integrating qualitative and quantitative data simultaneously	Variable experiences with staff and treatment Participants valued empathy and listening Lack of comfort and security in ED	Use and satisfaction with ED services	It included four models of ED Large sample size Integrative analysis Interview questions not provided
[51]	2004	Satisfaction with psychiatric services in the emergency department	Healthcare Policy	Mixed methods	Australia	*n* = 180 Participants who have presented to ED	Telephone interviews	Thematic analysis	High level of satisfac-tion with treatment in ED Long wait times Preference for staff with MH knowledge Inappropriate com-ments or treatment Chaotic environment in ED	Evaluation of people’s perceptions of and satisfaction with ED services	Large sample sizeSecondary analysis
[61]	2016	Patients’ experiences of psychiatric care in emergency departments: A secondary analysis	International Emergency Nursing	Qualitative Phenomenological method	USA	*n* = 9 Participants with a diagnosis of mental illness and previous experience accessing ED	Interviews and focus groups	Thematic analysis	Cold, clinical, and cha-otic physical environ-ment in ED Discriminatory treat-ment and feeling judged Loss of freedom Lack of privacy Long wait times Wanting staff with MH knowledge Mitigating effect of pos-itive interactions with staff	The primary aim is to describe the perceptions of ED visits by individuals experiencing a mental health crisis and identify themes to improve outcomes in ED settings	Secondary and comparative analysis of people who had also accessed an alternative to ED
[57]	2015	Patients with mental health issues in the emergency department: The relationship between coercion and perceptions of being helped, psychologically hurt and physically harmed	International Journal of Forensic Mental Health	Mixed methods	Canada	*n* = 49 Participants in an inpatient unit shortly after being triaged from the emergency department. Most involuntarily committed	Interviews Paper and pencil survey Likert and with open-ended questions	Thematic analysis of interview data	Mixed perceptions of treatment Lacking freedom Long wait times Importance of choice and shared decision making Treated differently Harmed by treatment	The aim was to understand how consumers perceived their experiences in the ED	Sampling bias, some people refused to participate because of the possible re-traumatisation Participants had all been transferred to the inpatient following ED presentation Most involun-tarily admis-sions
[70]	2019	To receive the patient in crisis with psychiatric emergencies: study of subjective experience	Medico-psychological Annals, psychiatric journal	Qualitative	Belgium	*n* = 12 Participants in ED	Semi-structured interviews	Thematic analysis	Waiting room was not welcoming Valued human and relational qualities of ED staff	To understand the subjective experience of patients of the waiting room	10 were first-time presentations
[66]	2012	Managing people with mental health presentations in emergency departments-a service exploration of the issues surrounding responsiveness from a mental health care consumer and carer perspective	Australasian Emergency Journal	mixed methods	Australia	*n* = 65 Survey *n*= 8 Focus group Participants who had utilised ED MH services in the previous six months and their families	Surveys and focus groups	Thematic analysis	ED necessary and ap-propriate Valued staff with MH knowledge Variable interactions with staff and treat-ment Unmet needs Long wait times Negative experiences in ED environment and wanting a separate MH space	Explore MH care in the ED specifically in relation to access and management	Small sample size in focus group Majority of participants female Limitations of satisfaction and dichotomous scaling Included consumers and carers Majority female
[71]	2009	Patient satisfaction with an emergency department psychiatric service	International Journal of Health Care Quality Assurance	Mixed methods	UK	*n* = 55 Participants who attended the ED	The Client Satisfaction Questionnaire Included two open-ended questions	Descriptive/thematic analysis	Variable experiences of staff and treatment Long wait times Lack of options Lack of information Communication	To measure the satisfaction of an emergency department psychiatric service	The response rate was low Only two open ended questions Positive bias in questions
[50]	2002	The quality of psychiatric services provided by an Australian tertiary hospital emergency department: a client perspective	Accident and Emergency Nursing	Mixed methods	Australia	*n*= 136 Participants who had to ED in the past six months	Telephone interviews	Thematic analysis	High level of satisfac-tion with service pro-vided by staff but some highly negative expe-riences Valued staff with MH knowledge	Evaluation of the inclusion of psychiatric nurse consultants	Focus on role of nurse consultants. Secondary analysis
[49]	2003	Patient satisfaction with psychiatric services	Journal of Psychiatric and Mental Health Nursing	Mixed methods	Australia	*n*= 136 Participants who had presented to the ED	Qualitative telephone interviews Structured questionnaire	Thematic/content coding of interview data	High level of satisfaction Valued staff with MH knowledge Long wait times Lack of privacy Inappropriate staff comments	Measure satisfaction with psychiatric services provided in a general ED	Results repre-sent same study by au-thors Secondary analysis
[62]	2018	Patient-centered values and experiences with emergency department and mental health crisis care	Administration and Policy in Mental Health	Qualitative	USA	*n* = 27 Participants who had received both psychiatric crisis care and community mental health services	Focus groups (*n =* 3)	Values-based coding	Not having basic needs met Negative experiences of involuntary treatment Valued communication and kindness Mitigating effect of positive staff interactions Valued shared decision making Wanted a quiet physical space	Explore consumers experiences and values about psychiatric care	A comparative study, some participants had received care in both ED and an alternative
[55]	2019	Why go to the emergency department? Perspectives from persons with borderline personality disorder	International Journal of Mental Health Nursing	Qualitative	Canada	*n* = 6 Participants diagnosed with borderline personality disorder with at least 12 ED visits with one year	Interviews	Thematic coding	ED was the only option Negative and discrim-inatory treatment Seeking connection Emotional impact of negative treatment	Understand the reasons why people with BPD go to the emergency and the perspective of persons with BPD	Focus on people with a diagnosis of BPD Small sample size Not generalisable Mostly women
[60]	2006	Consumer evaluation of a mental health liaison nurse service in the emergency department	Contemporary Nurse	Mixed-Method	Australia	*n* = 59 Participants who had accessed ED who had involvement with the M.H. liaison nurse while they were in the ED	phone survey/interviews	Coding	More positive experi-ence associated with having specialised MH staff MH nurses valued	Perceptions of treatment in ED	Focus on MHN One site. Limitations of satisfaction surveys.
[65]	2020	An inevitable response? A lived experience perspective on emergency responses to mental health crisis	Journal of Psychiatric Mental Health Nursing	A narrative account of personal experience	UK	*n* = 1 The individual experience of the author	Case study	Narrative analysis	Psychiatric pain is re-sponded to differently to physical pain Discriminatory treat-ment Criminalisation of MH	A narrative account of emergency pathways during psychiatric distress and potential impacts	Single individual Case study design
[54]	2017	“Hospital was the only option”: Experiences of frequent emergency department users in mental health	Administration and Policy in Mental Health	Mixed methods	USA	*n* = 20 Participants with diagnosis MI and addictions who frequently present to ED self-reported quantitative survey (*n* = 166)	In depth interviews survey	Thematic analysis	ED as appropriate/only option Mismatch of expecta-tions Discriminatory and unsatisfactory treat-ment Lack of follow up	To understand the experiences of service users who frequently present to ED	Frequent presentations to ED Includes an intervention group and treatment as usual
[58]	2020	Experiences of individuals who were physically restrained in the emergency department	JAMA Network Open	Qualitative	USA	*n* = 25 Participants who had been restrained in the ED	In-depth interviews	Thematic analysis	Harmful experiences of restraint Negative experiences with staff Lasting negative con-sequences of restraint	To characterise how individuals experience episodes of physical restraint during their ED visits	Majority male
[56]	2017	Don’t label me: A qualitative study of patients’ perceptions and experiences of sedation during behavioural emergencies in the emergency department.	Academic Emergency Medicine	Qualitative	Australia	*n* = 13 Participants who had received parenteral sedative medication	Face to face semi-structured interviews	Thematic analysis	Variable experiences of treatment Trust in staff Sedation seen as neces-sary Treated like a human being was important Long wait times led to distress Lack of debriefing and follow up Lack of information	Explore the perceptions and experiences of patients regarding the use of sedation during a mental health crisis	Small sample sizeConvenience sample Included par-ticipants who presented for the first time

## References

[1] Akther S.F., Molyneaux E., Stuart R., Johnson S., Simpson A., Oram S. (2019). Patients’ experiences of assessment and detention under mental health legislation: Systematic review and qualitative meta-synthesis. BJPsych Open.

[2] Henderson J. (2005). Neo-liberalism, community care and Australian mental health policy. Health Sociol. Rev..

[3] Shen G.C., Eaton J., Snowden L. (2017). Mainstreaming Mental Health Care in 42 Countries. Health Syst. Reform.

[4] Marynowski-Traczyk D., Moxham L., Broadbent M. (2013). A critical discussion of the concept of recovery for mental health consumers in the Emergency Department. Australas. Emerg. Nurs. J..

[5] Roggenkamp R., Andrew E., Nehme Z., Cox S., Smith K. (2018). Descriptive Analysis of Mental Health-Related Presentations To Emergency Medical Services. Prehospital Emerg. Care.

[6] Larkin G.L., Beautrais A.L., Spirito A., Kirrane B.M., Lippmann M.J., Milzman D. (2009). Mental Health and Emergency Medicine: A Research Agenda. Acad. Emerg. Med..

[7] American College of Emergency Physicians Mental Health Advocacy. https://www.acep.org/federal-advocacy/mental-health/.

[8] Australian Institute of Health and Welfare (2017). Emergency Department Care 2016–2017: Australian Hospital Statistics.

[9] Capp R., Hardy R., Lindrooth R., Wiler J. (2016). National Trends in Emergency Department Visits by Adults with Mental Health Disorders. J. Emerg. Med..

[10] Australian Institute of Health and Welfare (2021). Mental Health Services in Australia.

[11] Barratt H., García A.R., Clarke K., Moore A., Whittington C., Stockton S., Thomas J., Pilling S., Raine R. (2016). Epidemiology of Mental Health Attendances at Emergency Departments: Systematic Review and Meta-Analysis. PLoS ONE.

[12] Theriault K.M., Rosenheck R.A., Rhee T.G. (2020). Increasing Emergency Department Visits for Mental Health Conditions in the United States. J. Clin. Psychiatry.

[13] Care Quality Commission (2015). Right Here, Right Now: People’s Experiences of Help, Care and Support. During a Mental Health Crisis.

[14] Ceniti A.K., Heinecke N., McInerney S.J. (2018). Examining suicide-related presentations to the emergency department. Gen. Hosp. Psychiatry.

[15] Nordstrom K., Berlin J.S., Nash S.S., Shah S.B., Schmelzer N.A., Worley L.L. (2019). Boarding of Mentally Ill Patients in Emergency Departments: American Psychiatric Association Resource Document. West. J. Emerg. Med..

[16] Nicks B.A., Manthey D.M. (2012). The Impact of Psychiatric Patient Boarding in Emergency Departments. Emerg. Med. Int..

[17] Appelbaum P.S. (2015). “Boarding” Psychiatric Patients in Emergency Rooms: One Court Says “No More”. Psychiatr. Serv..

[18] Australasian College for Emergency Medicine (2018). The Long Wait: An Analysis of Mental Health Presentations to Australian Emergency Departments. https://acem.org.au/getmedia/60763b10-1bf5-4fbc-a7e2-9fd58620d2cf/ACEM_report_41018.

[19] Allison S., Bastiampillai T., O’Reilly R., Sharfstein S.S., Castle D. (2019). Widespread emergency department access block: A human rights issue in Australia?. Australas. Psychiatry.

[20] Knott J., Gerdtz M., Dobson S., Daniel C., Graudins A., Mitra B., Bartley B., Chapman P. (2019). Restrictive interventions in Victorian emergency departments: A study of current clinical practice. Emerg. Med. Australas..

[21] McKenna B., Furness T., Maguire T. (2014). A Literature Review and Policy Analysis on the Practice of Restrictive Interventions.

[22] HMICFRS (2018). Policing and Mental Health Picking Up the Pieces. https://www.justiceinspectorates.gov.uk/hmicfrs/wp-content/uploads/policing-and-mental-health-picking-up-the-pieces.pdf.

[23] Waldemar A.K., Arnfred S.M., Petersen L., Korsbek L. (2016). Recovery-Oriented Practice in Mental Health Inpatient Settings: A Literature Review. Psychiatr. Serv..

[24] Van Weeghel J., van Zelst C., Boertien D., Hasson-Ohayon I. (2019). Conceptualizations, assessments, and implications of personal recovery in mental illness: A scoping review of systematic reviews and meta-analyses. Psychiatr. Rehabil. J..

[25] Le Boutillier C., Leamy M., Bird V., Davidson L., Williams J., Slade M. (2011). What Does Recovery Mean in Practice? A Qualitative Analysis of International Recovery-Oriented Practice Guidance. Psychiatr. Serv..

[26] Oades L.G., Anderson J. (2012). Recovery in Australia: Marshalling strengths and living values. Int. Rev. Psychiatry.

[27] Martinelli A., Ruggeri M. (2020). An overview of mental health recovery-oriented practices: Potentiality, challenges, prejudices, and misunderstandings. J. Psychopathol..

[28] Deacon B.J. (2013). The biomedical model of mental disorder: A critical analysis of its validity, utility, and effects on psychotherapy research. Clin. Psychol. Rev..

[29] Davidson L. (2020). A Key, not a Straitjacket: The Case for Interim Mental Health Legislation Pending Complete Prohibition of Psychiatric Coercion in Accordance with the Convention on the Rights of Persons with Disabilities. Health Hum. Rights.

[30] Boscarato K., Lee S.J., Kroschel J., Hollander Y., Brennan A., Warren N. (2014). Consumer experience of formal crisis-response services and preferred methods of crisis intervention. Int. J. Ment. Health Nurs..

[31] Carstensen K., Lou S., Jensen L.G., Nissen N.K., Ortenblad L., Pfau M., Ankersen P.V. (2017). Psychiatric service users’ experiences of emergency departments: A CERQual review of qualitative studies. Nord. J. Psychiatry.

[32] Hobbs M. (1984). Crisis intervention in theory and practice: A selective review. Br. J. Med. Psychol..

[33] Ball J.S., Links P.S., Strike C., Boydell K. (2005). “It’s overwhelming. Everything seems to be too much”: A theory of crisis for individuals with severe persistent mental illness. Psychiatr. Rehabil. J..

[34] Borschmann R., Henderson C., Hogg J., Phillips R., Moran P. (2012). Crisis interventions for people with borderline personality disorder. Cochrane Database Syst. Rev..

[35] Arksey H., O’Malley L. (2005). Scoping studies: Towards a methodological framework. Int. J. Soc. Res. Methodol..

[36] Colquhoun H. (2016). Current Best Practices for the Conduct of Scoping Reviews. https://www.equator-network.org/wp-content/uploads/2016/06/Gerstein-Library-scoping-reviews_May-12.pdf.

[37] Tricco A.C., Lillie E., Zarin W., O’Brien K.K., Colquhoun H., Levac D., Moher D., Peters M.D.J., Horsley T., Weeks L. (2018). PRISMA extension for scoping reviews (PRISMA-ScR): Checklist and explanation. Ann. Intern. Med..

[38] Mays N., Pope C., Popay J. (2005). Systematically reviewing qualitative and quantitative evidence to inform management and policy-making in the health field. J. Health Serv. Res. Policy.

[39] Zomerdijk L.G., Voss C. (2009). Service Design for Experience-Centric Services. J. Serv. Res..

[40] Moher D., Liberati A., Tetzlaff J., Altman D.G. (2009). The PRISMA Group Preferred Reporting Items for Systematic Reviews and Meta-Analyses: The PRISMA Statement. PLoS Med..

[41] Perreault M., Pawliuk N., Veilleux R., Rousseau M. (2006). Qualitative Assessment of Mental Health Service Satisfaction: Strengths and Limitations of a Self-Administered Procedure. Community Ment. Health J..

[42] Williams B., Coyle J., Healy D. (1998). The meaning of patient satisfaction: An explanation of high reported levels. Soc. Sci. Med..

[43] Thomas D. (2006). A General Inductive Approach for Analyzing Qualitative Evaluation Data. Am. J. Eval..

[44] Glaser B., Strauss A.L. (1967). The Discovery of Grounded Theory: Strategies for Qualitative Research.

[45] DeSantis L., Ugarriza D.N. (2000). The concept of theme as used in qualitative nursing research. West. J. Nurs. Res..

[46] Ryan G.W., Bernard H.R. (2003). Techniques to Identify Themes. Field Methods.

[47] Samson S., Granath K., Alger A. (2017). Journey Mapping the User Experience. Coll. Res. Libr..

[48] Page M.J., McKenzie J.E., Bossuyt P.M., Boutron I., Hoffmann T.C., Mulrow C.D., Shamseer L., Tetzlaff J.M., Akl E.A., Brennan S.E. (2021). The PRISMA 2020 statement: An updated guideline for reporting systematic reviews. BMJ.

[49] Summers M., Happell B. (2003). Patient satisfaction with psychiatric services provided by a Melbourne tertiary hospital emergency department. J. Psychiatr. Ment. Health Nurs..

[50] Summers M., Happell B. (2002). The quality of psychiatric services provided by an Australian tertiary hospital emergency department: A client perspective. Accid. Emerg. Nurs..

[51] Happell B., Summers M. (2004). Satisfaction with psychiatric services in the emergency department. Int. Psychiatry.

[52] Cerel J., Currier G.W., Conwell Y. (2006). Consumer and Family Experiences in the Emergency Department Following a Suicide Attempt. J. Psychiatr. Pr..

[53] VanDyk A.D., Young L., Macphee C., Gillis K. (2017). Exploring the Experiences of Persons Who Frequently Visit the Emergency Department for Mental Health-Related Reasons. Qual. Health Res..

[54] Wise-Harris D., Pauly D., Kahan D., De Bibiana J.T., Hwang S.W., Stergiopoulos V. (2016). “Hospital was the Only Option”: Experiences of Frequent Emergency Department Users in Mental Health. Adm. Policy Ment. Health Ment. Health Serv. Res..

[55] Vandyk A., Bentz A., Bissonette S., Cater C. (2019). Why go to the emergency department? Perspectives from persons with borderline personality disorder. Int. J. Ment. Health Nurs..

[56] Yap C.Y.L., Knott J.C., Kong D.C.M., Gerdtz M., Stewart K., Taylor D.M. (2017). Don’t Label Me: A Qualitative Study of Patients’ Perceptions and Experiences of Sedation During Behavioral Emergencies in the Emergency Department. Acad. Emerg. Med..

[57] Harrison N., Mordell S., Roesch R., Watt K. (2015). Patients with Mental Health Issues in the Emergency Department: The Relationship Between Coercion and Perceptions of Being Helped, Psychologically Hurt, and Physically Harmed. Int. J. Forensic Ment. Health.

[58] Wong A.H., Ray J.M., Rosenberg A., Crispino L., Parker J., McVaney C., Iennaco J.D., Bernstein S.L., Pavlo A.J. (2020). Experiences of Individuals Who Were Physically Restrained in the Emergency Department. JAMA Netw. Open.

[59] Eales S., Callaghan P., Johnson B. (2006). Service users and other stakeholders’ evaluation of a liaison mental health service in an accident and emergency department and a general hospital setting. J. Psychiatr. Ment. Health Nurs..

[60] Wand T., Schaecken P. (2006). Consumer evaluation of a mental health liaison nurse service in the Emergency Department. Contemp. Nurse.

[61] Harris B., Beurmann R., Fagien S., Shattell M. (2015). Patients’ experiences of psychiatric care in emergency departments: A secondary analysis. Int. Emerg. Nurs..

[62] Thomas K.C., Owino H., Ansari S., Adams L., Cyr J.M., Gaynes B.N., Glickman S.W. (2018). Patient-Centered Values and Experiences with Emergency Department and Mental Health Crisis Care. Adm. Policy Ment. Health Ment. Health Serv. Res..

[63] Fleury M.-J., Grenier G., Farand G.G.A.L. (2019). Satisfaction with Emergency Departments and Other Mental Health Services among Patients with Mental Disorders. Health Policy.

[64] Carpenter L.L., Schecter J.M., Underwood J.A., Tyrka A.R., Price L.H. (2005). Service Expectations and Clinical Characteristics of Patients Receiving Psychiatric Emergency Services. Psychiatr. Serv..

[65] White C.J. (2020). An inevitable response? A lived experienced perspective on emergency responses to mental health crises. J. Psychiatr. Ment. Health Nurs..

[66] Morphet J., Innes K., Munro I., O’Brien A., Gaskin C.J., Reed F., Kudinoff T. (2012). Managing people with mental health presentations in emergency departments—A service exploration of the issues surrounding responsiveness from a mental health care consumer and carer perspective. Australas. Emerg. Nurs. J..

[67] Allen M.H., Carpenter D., Sheets J.L., Miccio S., Ross R. (2003). What do consumers say they want and need during a psychiatric emergency?. J. Psychiatr. Pract..

[68] Clarke D.E., Dusome D., Hughes L. (2007). Emergency department from the mental health client’s perspective. Int. J. Ment. Health Nurs..

[69] Callaghan P., Eales S., Coats T., Bowers L., Bunker J. (2002). Patient feedback on liaison mental health care in A&E. Nurs. Times.

[70] Meriaux M., Denis J., Michel V., Hendrick S. (2019). To receive the patient in crisis in psychiatric emergencies: Subjective experience study. Ann. Med.-Psychol..

[71] O’Regan C., Ryan M. (2009). Patient satisfaction with an emergency department psychiatric service. Int. J. Health Care Qual. Assur..

[72] Duggan M., Harris B., Chislett W.-K., Calder R. (2020). Nowhere Else to Go: Why Australia’s Health System Results in People with Mental Illness Getting ‘Stuck’ in Emergency Departments. https://www.vu.edu.au/sites/default/files/nowhere-else-to-go-people-mental-illness-stuck-emergency-departments-report-mitchell-institute.pdf..

[73] Unwin M., Crisp E., Stankovich J., McCann D., Kinsman L. (2020). Socioeconomic disadvantage as a driver of non-urgent emergency department presentations: A retrospective data analysis. PLoS ONE.

[74] Ferguson N., Savic M., McCann T.V., Emond K., Sandral E., Smith K., Roberts L., Bosley E., Lubman D.I. (2019). “I was worried if I don’t have a broken leg, they might not take it seriously”: Experiences of men accessing ambulance services for mental health and/or alcohol and other drug problems. Health Expect..

[75] Repper J. (1995). The deserving and the undeserving: Selectivity and progress in a community care service. J. Ment. Health.

[76] Olthuis G., Prins C., Smits M.-J., van de Pas H., Bierens J., Baart A. (2014). Matters of Concern: A Qualitative Study of Emergency Care from the Perspective of Patients. Ann. Emerg. Med..

[77] Innes K., Morphet J., O’Brien A.P., Munro I. (2014). Caring for the mental illness patient in emergency departments–an exploration of the issues from a healthcare provider perspective. J. Clin. Nurs..

[78] Broadbent M., Moxham L., Dwyer T. (2014). Implications of the emergency department triage environment on triage practice for clients with a mental illness at triage in an Australian context. Australas. Emerg. Nurs. J..

[79] Gulrajani R. (1995). Physical environmental factors affecting factors affecting patients’ stress in the accident and emergency department. Accid. Emerg. Nurs..

[80] Greenfield T.K., Stoneking B.C., Humphreys K., Sundby E., Bond J. (2008). A Randomized Trial of a Mental Health Consumer-Managed Alternative to Civil Commitment for Acute Psychiatric Crisis. Am. J. Community Psychol..

[81] Kerrison S.A., Chapman R. (2007). What general emergency nurses want to know about mental health patients presenting to their emergency department. Accid. Emerg. Nurs..

[82] Ray J.D., Overman A.S. (2014). Hard facts about soft skills. AJN Am. J. Nurs..

[83] Kumble S., McSherry B. (2010). Seclusion and restraint: Rethinking regulation from a human rights perspective. Psychiatry Psychol. Law.

[84] WHO (2021). Guidance on Community Mental Health Services: Promoting Person-Centred and Rights-Based Approaches.

[85] Al-Maraira O.A., Hayajneh F.A. (2019). Use of Restraint and Seclusion in Psychiatric Settings: A Literature Review. J. Psychosoc. Nurs. Ment. Health Serv..

[86] Brophy L.M., Roper C.E., Hamilton B.E., Tellez J.J., McSherry B.M. (2016). Consumers and their supporters’ perspectives on poor practice and the use of seclusion and restraint in mental health settings: Results from Australian focus groups. Int. J. Ment. Health Syst..

[87] Bombard Y., Baker G.R., Orlando E., Fancott C., Bhatia P., Casalino S., Onate K., Denis J.-L., Pomey M.-P. (2018). Engaging patients to improve quality of care: A systematic review. Implement. Sci..

[88] Australian Government Department of Health and Ageing (2013). National Mental Health Report 2013: Tracking Progress of Mental Health Reform in Australia 1993–2011.

[89] Hall A.E., Bryant J., Sanson-Fisher R., Fradgley E.A., Proietto A.M., Roos I. (2018). Consumer input into health care: Time for a new active and comprehensive model of consumer involvement. Health Expect..

[90] National Mental Health Commission (2019). Monitoring Mental Health and Suicide Prevention Reform: National Report 2019.

[91] NHS (2019). NHS Mental Health Implementation Plan. 2019/20-2023/24.

